# Supplementary material for: Analysis of PPARγ Signaling Activity in Psoriasis
Source: Int J Mol Sci. 2021 Aug 10;22(16):8603. doi: 10.3390/ijms22168603 (PMC8395241; doi:10.3390/ijms22168603)
Supplement: Supplementary file 1 [file ijms-22-08603-s001.zip › Supplemental materials_Analysis of PPARg signaling activity in psoriasis/Pathway models/Models images and html files/Model of PPARG related pathways in psoriasis (short version)/445.html]

AHR


# Protein AHR

|  |  |
| --- | --- |
| URN | urn:agi-llid:196 |
| Total Entities | 0 |
| Connectivity | 4587 |
| Name | AHR |
| Description | aryl hydrocarbon receptor |
| Notes | The protein encoded by this gene is a ligand-activated helix-loop-helix transcription factor involved in the regulation of biological responses to planar aromatic hydrocarbons. This receptor has been shown to regulate xenobiotic-metabolizing enzymes such as cytochrome P450. Before ligand binding, the encoded protein is sequestered in the cytoplasm; upon ligand binding, this protein moves to the nucleus and stimulates transcription of target genes. [provided by RefSeq, Sep 2015] |
| Primary Cell Localization | Nucleus |
| Class | Transcription factor |

---

|  |  |
| --- | --- |
| Pathway | Longevity Related Drugs |
|  | AHR Signaling in Treg and Dendritic Cells Function |
|  | Kynurenine/AHR Signaling in Treg Cell Activation |
|  | AHR Signaling in Th17 Cells Function |
|  | AHR Signaling in Tr1 Cells Function |
|  | AHR in Intestinal Cell Antimicrobial Barrier Maintenance |
|  | AHR Signaling in M1 Macrophages Function |
|  | Hematopoietic Cell Lineage: B-cell |
|  | Proteins Involved in Arterial Hypertension |
|  | Proteins Involved in Vitiligo |
|  | Proteins Involved in non-Alcoholic Fatty Liver Disease |
|  | Proteins Involved in Glioma |
|  | Proteins Involved in Endometriosis |
|  | Dioxin Role in Endometriosis |
|  | Aryl Hydrocarbon Receptor/Tryptophan Metabolites Signaling |
|  | Aryl Hydrocarbon Receptor Genomic and non-Genomic Signaling |
|  | Aryl Hydrocarbon Receptor/Arachidonic Acid Metabolites Signaling |
|  | Aryl Hydrocarbon Receptor/Heme-Derived Molecules Signaling |
|  | Ubiquitin Ligase Complex in Prostata Cell |
|  | Treg Cells Promote Immunosuppression in Cancer Immune Escape |
|  | IDO1 in Cancer Immune Escape |
|  | CD40 -> Expression Targets in Lymph Node |
|  | Chemokine Receptor Family -> Expression Targets in Lymphoid System |
|  | ER Stress (Unfolded Protein Response) |
|  | Oxidative Stress Protection via AHR-NFE2L2-NQO1 Signaling in Keratinocytes |
|  | Dioxin Induced Chloracne (Hypothesis) |
|  | B-Cell Differentiation Inhibition by Dioxin |
|  | prarg negative regulators, ps-positive |
|  | PPARG negative regulators |
|  | pparg expr target\_regulators |
|  | Neighbors of disease exacerbation |
|  | Neighbors of tumor progression |
|  | Neighbors of cancer progression |
|  | Neighbors of inflammatory status |
|  | Neighbors of overall survival |
|  | Neighbors of disease severity |
|  | Neighbors of tumor size |
|  | Neighbors of mortality |
|  | Neighbors of survival rate |
|  | Neighbors of survival time |
|  | Neighbors of insulin sensitivity |
|  | Neighbors of bacterial load |
|  | Neighbors of cancer survival |
|  | Neighbors of graft survival |
|  | Neighbors of therapeutic efficacy |
|  | Neighbors of clinical stage |
|  | Neighbors of lethality |
|  | Neighbors of recurrence-free survival |
|  | Neighbors of treatment outcome |
|  | Neighbors of bone mass |
|  | Neighbors of body weight |
|  | Neighbors of body weight gain |
|  | Neighbors of cardiovascular risk |
|  | Neighbors of tumor promotion |
|  | Neighbors of glucose tolerance |
|  | Neighbors of morbidity |
|  | Neighbors of exhaustion |
|  | Neighbors of bone mineral density |
|  | Neighbors of adverse outcome |
|  | Neighbors of radioresistance |
|  | Neighbors of apoptosis rate |
|  | Neighbors of shear stress |
|  | Neighbors of blood pressure |
|  | Neighbors of leukocyte count |
|  | Neighbors of tumor grade |
|  | Neighbors of cancer risk |
|  | Neighbors of cancer incidence |
|  | Neighbors of tumor aggressiveness |
|  | Neighbors of systolic blood pressure |
|  | Neighbors of ejection fraction |
|  | Neighbors of fasting plasma glucose |
|  | Neighbors of T-cell count |
|  | Neighbors of energy expenditure |
|  | Neighbors of glucose level |
|  | Neighbors of breast cancer risk |
|  | Neighbors of proliferation index |
|  | Neighbors of pregnancy outcome |
|  | Neighbors of gastric cancer risk |
|  | Neighbors of fertility |
|  | Neighbors of pathological response |
|  | Neighbors of lymphocyte count |
|  | Neighbors of metabolic response |
|  | Neighbors of fibroblast phenotype |
|  | Neighbors of fractional shortening |
|  | Neighbors of disease exacerbation |
|  | Neighbors of tumor progression |
|  | Neighbors of cancer progression |
|  | Neighbors of inflammatory status |
|  | Neighbors of overall survival |
|  | Neighbors of disease severity |
|  | Neighbors of tumor size |
|  | Neighbors of mortality |
|  | Neighbors of survival rate |
|  | Neighbors of survival time |
|  | Neighbors of insulin sensitivity |
|  | Neighbors of bacterial load |
|  | Neighbors of cancer survival |
|  | Neighbors of graft survival |
|  | Neighbors of therapeutic efficacy |
|  | Neighbors of clinical stage |
|  | Neighbors of lethality |
|  | Neighbors of recurrence-free survival |
|  | Neighbors of treatment outcome |
|  | Neighbors of bone mass |
|  | Neighbors of body weight |
|  | Neighbors of body weight gain |
|  | Neighbors of cardiovascular risk |
|  | Neighbors of tumor promotion |
|  | Neighbors of glucose tolerance |
|  | Neighbors of morbidity |
|  | Neighbors of exhaustion |
|  | Neighbors of bone mineral density |
|  | Neighbors of adverse outcome |
|  | Neighbors of radioresistance |
|  | Neighbors of apoptosis rate |
|  | Neighbors of shear stress |
|  | Neighbors of blood pressure |
|  | Neighbors of leukocyte count |
|  | Neighbors of tumor grade |
|  | Neighbors of cancer risk |
|  | Neighbors of cancer incidence |
|  | Neighbors of tumor aggressiveness |
|  | Neighbors of systolic blood pressure |
|  | Neighbors of ejection fraction |
|  | Neighbors of fasting plasma glucose |
|  | Neighbors of T-cell count |
|  | Neighbors of energy expenditure |
|  | Neighbors of glucose level |
|  | Neighbors of breast cancer risk |
|  | Neighbors of proliferation index |
|  | Neighbors of pregnancy outcome |
|  | Neighbors of gastric cancer risk |
|  | Neighbors of fertility |
|  | Neighbors of pathological response |
|  | Neighbors of lymphocyte count |
|  | Neighbors of metabolic response |
|  | Neighbors of fibroblast phenotype |
|  | Neighbors of fractional shortening |
|  | Neighbors of cell development |
|  | Neighbors of immune response |
|  | Neighbors of cell population |
|  | Neighbors of T-cell development |
|  | Neighbors of inflammatory response |
|  | Neighbors of wound healing |
|  | Neighbors of angiogenesis |
|  | Neighbors of innate immune response |
|  | Neighbors of cell function |
|  | Neighbors of macrophage activation |
|  | Neighbors of cell formation |
|  | Neighbors of adaptive immune response |
|  | Neighbors of osteoclast development |
|  | Neighbors of epithelial to mesenchymal transition |
|  | Neighbors of immunity |
|  | Neighbors of cell proliferative response |
|  | Neighbors of chemotaxis |
|  | Neighbors of dendritic cell differentiation |
|  | Neighbors of cell survival |
|  | Neighbors of cell phenotype |
|  | Neighbors of T-cell proliferation |
|  | Neighbors of tissue remodeling |
|  | Neighbors of cell adhesion |
|  | Neighbors of T-cell activation |
|  | Neighbors of fibrogenesis |
|  | Neighbors of tumor growth |
|  | Neighbors of dendritic cell development |
|  | Neighbors of tissue repair |
|  | Neighbors of cell count |
|  | Neighbors of cell infiltration |
|  | Neighbors of cell interaction |
|  | Neighbors of lumen formation |
|  | Neighbors of endothelial cell migration |
|  | Neighbors of adipogenesis |
|  | Neighbors of lipid storage |
|  | Neighbors of cell damage |
|  | Neighbors of pregnancy |
|  | Neighbors of monocyte differentiation |
|  | Neighbors of aging |
|  | Neighbors of cell motility |
|  | Neighbors of bone resorption |
|  | Neighbors of monocyte migration |
|  | Neighbors of fibroblast proliferation |
|  | Neighbors of adipocyte differentiation |
|  | Neighbors of microglial activation |
|  | Neighbors of neutrophil recruitment |
|  | Neighbors of regeneration |
|  | Neighbors of cell migration |
|  | Neighbors of cancer growth |
|  | Neighbors of stem cell proliferation |
|  | Neighbors of hepatic regeneration |
|  | Neighbors of phagocytosis |
|  | Neighbors of immune system activation |
|  | Neighbors of leukocyte migration |
|  | Neighbors of leukocyte recruitment |
|  | Neighbors of osteoclast formation |
|  | Neighbors of monocyte recruitment |
|  | Neighbors of cell invasion |
|  | Neighbors of heart function |
|  | Neighbors of hemopoiesis |
|  | Neighbors of bone remodeling |
|  | Neighbors of ossification |
|  | Neighbors of T-cell function |
|  | Neighbors of immune system function |
|  | Neighbors of lung development |
|  | Neighbors of autophagy |
|  | Neighbors of transcription activation |
|  | Neighbors of senescence |
|  | Neighbors of T-cell response |
|  | Neighbors of tumor immunity |
|  | Neighbors of hepatocyte proliferation |
|  | Neighbors of stem cell differentiation |
|  | Neighbors of endothelial cell function |
|  | Neighbors of neuronal death |
|  | Neighbors of cell growth |
|  | Neighbors of ROS generation |
|  | Neighbors of epithelial cell proliferation |
|  | Neighbors of cancer cell growth |
|  | Neighbors of sensitization |
|  | Neighbors of hemato-encephalic barrier |
|  | Neighbors of macrophage function |
|  | Neighbors of cellular immune response |
|  | Neighbors of cellular senescence |
|  | Neighbors of colony formation |
|  | Neighbors of macrophage differentiation |
|  | Neighbors of cell differentiation |
|  | Neighbors of stem cell migration |
|  | Neighbors of life span |
|  | Neighbors of cell homeostasis |
|  | Neighbors of psoriasis |
|  | Neighbors of inflammatory disease |
|  | Neighbors of rheumatoid arthritis |
|  | Neighbors of atherosclerosis |
|  | Neighbors of fibrosis |
|  | Neighbors of colitis |
|  | Neighbors of arthritis |
|  | Neighbors of inflammatory bowel disease |
|  | Neighbors of injury |
|  | Neighbors of atherogenesis |
|  | Neighbors of vascular remodeling |
|  | Neighbors of metastasis |
|  | Neighbors of carcinogenesis |
|  | Neighbors of multiple sclerosis |
|  | Neighbors of chronic inflammation |
|  | Neighbors of inflammation |
|  | Neighbors of tumor microenvironment |
|  | Neighbors of obesity |
|  | Neighbors of diabetes mellitus |
|  | Neighbors of asthma |
|  | Neighbors of pneumonia |
|  | Neighbors of hyperplasia |
|  | Neighbors of liver fibrosis |
|  | Neighbors of chronic obstructive pulmonary disease |
|  | Neighbors of leukocyte infiltration |
|  | Neighbors of reperfusion injury |
|  | Neighbors of airway inflammation |
|  | Neighbors of insulin resistance |
|  | Neighbors of infection |
|  | Neighbors of vascular injury |
|  | Neighbors of renal injury |
|  | Neighbors of liver injury |
|  | Neighbors of neuroinflammation |
|  | Neighbors of death |
|  | Neighbors of encephalomyelitis |
|  | Neighbors of autoimmune disease |
|  | Neighbors of dermatitis |
|  | Neighbors of neutrophil infiltration |
|  | Neighbors of heart disease |
|  | Neighbors of lung injury |
|  | Neighbors of immunopathology |
|  | Neighbors of hepatocellular carcinoma |
|  | Neighbors of autoimmunity |
|  | Neighbors of gastric cancer |
|  | Neighbors of atherosclerotic plaque |
|  | Neighbors of experimental autoimmune encephalomyelitis |
|  | Neighbors of colorectal cancer |
|  | Neighbors of breast cancer |
|  | Neighbors of ischemia |
|  | Neighbors of systemic lupus erythematosus |
|  | Neighbors of melanoma |
|  | Neighbors of collagen-induced arthritis |
|  | Neighbors of cartilage degeneration |
|  | Neighbors of neoplasm invasion |
|  | Neighbors of kidney disease |
|  | Neighbors of lung disease |
|  | Neighbors of cardiovascular disease |
|  | Neighbors of cancer |
|  | Neighbors of type 2 diabetes |
|  | Neighbors of neoplasm |
|  | Neighbors of endometriosis |
|  | Neighbors of vasculitis |
|  | Neighbors of atrophy |
|  | Neighbors of diet-induced obesity |
|  | Neighbors of respiratory hypersensitivity |
|  | Neighbors of myocardial fibrosis |
|  | Neighbors of synovitis |
|  | Neighbors of endothelial cell dysfunction |
|  | Neighbors of liver disease |
|  | Neighbors of prostate cancer |
|  | Neighbors of hypertrophy |
|  | Neighbors of osteolysis |
|  | Neighbors of ovarian cancer |
|  | Neighbors of pancreatic cancer |
|  | Neighbors of hypertension |
|  | Neighbors of hepatocyte |
|  | Neighbors of mesenchymal stroma cell |
|  | Neighbors of stem cell |
|  | Neighbors of B-cell |
|  | Neighbors of T-cell |
|  | Neighbors of lung |
|  | Neighbors of liver |
|  | Neighbors of skin |
|  | Neighbors of intestine |
|  | Neighbors of lymph node |
|  | Neighbors of gastrointestinal tract |
|  | Neighbors of bone |
|  | Neighbors of ovary |
|  | Neighbors of breast |
|  | Neighbors of cardiovascular system |
|  | Neighbors of immune system |
|  | Neighbors of myelin sheath |
|  | Neighbors of nervous system |
|  | Neighbors of endocrine system |
|  | Neighbors of reproductive system |
|  | Neighbors of NF-kB family |
|  | Neighbors of IL1 family |
|  | Neighbors of Jun/Fos |
|  | Neighbors of PI3K |
|  | Neighbors of STAT family |
|  | Neighbors of mitogen-activated protein kinase |
|  | Neighbors of IFNAR ligand |
|  | Neighbors of TLR |
|  | Neighbors of interferon |
|  | Neighbors of PDGF |
|  | Neighbors of growth factor |
|  | Neighbors of PKA |
|  | Neighbors of chorionic gonadotropin |
|  | Neighbors of protein tyrosine kinase |
|  | Neighbors of SMAD subfamily |
|  | Neighbors of LXR |
|  | Neighbors of IL23 |
|  | Neighbors of heat shock protein 90 |
|  | Neighbors of proteasome endopeptidase complex |
|  | Neighbors of PP2A |
|  | Neighbors of retinoid-X receptor subfamily |
|  | Neighbors of WNT |
|  | Neighbors of gonadotropins |
|  | Neighbors of FSH |
|  | Neighbors of RA receptor |
|  | Neighbors of histone |
|  | Neighbors of PDGF receptor |
|  | Neighbors of phosphodiesterase |
|  | Model of PPARG signaling in psoriasis |
|  | PPARG negative regulators and targets |
|  | Model of PPARG related pathways in psoriasis (short version) |
|  | 2\_Defective tolerance of autoreactive T-cell in T1D |
|  | drug-target pairs direct regulation |
|  | drug-target molsynthesis |

---

|  |  |
| --- | --- |
| MedScan ID | 196 |

---

|  |  |
| --- | --- |
| LocusLink ID | 196 |
|  | 11622 |
|  | 25690 |

---

|  |  |
| --- | --- |
| Alias | LOC193333 |
|  | Ah receptor |
|  | dioxin receptor |
|  | Ah receptors |
|  | aromatic hydrocarbon receptor |
|  | AHR\_HUMAN |
|  | class E basic helix-loop-helix protein 76 |
|  | AhR |
|  | dioxin receptors |
|  | bHLHe76 |
|  | aromatic hydrocarbon responsiveness |
|  | AHR2 |
|  | aryl hydrocarbon receptors |
|  | Ahre |
|  | aryl hydrocarbon receptor |
|  | RP85 |
|  | AH-receptor |
|  | Ah |
|  | In |
|  | Ahh |

---

|  |  |
| --- | --- |
| GO ID | 0003677 |
|  | 0003700 |
|  | 0000981 |
|  | 0070888 |
|  | 0051879 |
|  | 0017025 |
|  | 0001094 |
|  | 0035326 |
|  | 0004879 |
|  | 0046982 |
|  | 0042803 |
|  | 1990837 |
|  | 0001223 |
|  | 0008134 |
|  | 0044212 |
|  | 0006915 |
|  | 0001568 |
|  | 0019933 |
|  | 0007049 |
|  | 1904613 |
|  | 0071320 |
|  | 1904322 |
|  | 0032922 |
|  | 0030522 |
|  | 0045892 |
|  | 0045944 |
|  | 0045893 |
|  | 0030888 |
|  | 0010468 |
|  | 0006357 |
|  | 0006355 |
|  | 0009636 |
|  | 0009410 |
|  | 0006366 |
|  | 0006805 |
|  | 0005737 |
|  | 0005829 |
|  | 0034752 |
|  | 0005654 |
|  | 0005634 |
|  | 0032991 |
|  | 0005667 |
|  | 0017162 |
|  | 0051087 |
|  | 0043565 |
|  | 0015643 |
|  | 0030183 |
|  | 0001782 |
|  | 0001922 |
|  | 0043029 |
|  | 0008015 |
|  | 0048514 |
|  | 0001974 |
|  | 0001569 |
|  | 0043010 |
|  | 0003214 |
|  | 0000902 |
|  | 1904682 |
|  | 0003243 |
|  | 0061009 |
|  | 0035162 |
|  | 0048732 |
|  | 0072102 |
|  | 0002376 |
|  | 0060993 |
|  | 0001889 |
|  | 0002260 |
|  | 2000279 |
|  | 0010693 |
|  | 1903170 |
|  | 0060547 |
|  | 0045668 |
|  | 0033689 |
|  | 0003085 |
|  | 0000122 |
|  | 0045906 |
|  | 0001541 |
|  | 0045899 |
|  | 0045793 |
|  | 0040010 |
|  | 0035166 |
|  | 0030850 |
|  | 0008217 |
|  | 0097746 |
|  | 0060420 |
|  | 0048608 |
|  | 0014070 |
|  | 0048511 |
|  | 0048745 |
|  | 0048536 |
|  | 0060841 |
|  | 0034751 |
|  | 0034753 |
|  | 0071385 |
|  | 0051726 |
|  | 0032355 |
|  | 0004874 |
|  | 0003705 |
|  | 0050880 |
|  | 0006950 |
|  | 0006351 |
|  | 0005730 |
|  | 0004871 |
|  | 0007165 |
|  | 0010843 |
|  | 0030528 |
|  | 0004872 |
|  | 0045927 |

---

|  |  |
| --- | --- |
| KEGG ID | hsa:196 |
|  | mmu:11622 |
|  | rno:25690 |

---

|  |  |
| --- | --- |
| Organism | Homo sapiens {Organism urn:agi-taxid:9606} |
|  | Mus musculus {Organism urn:agi-taxid:10090} |
|  | Rattus norvegicus {Organism urn:agi-taxid:10116} |
|  | Homo sapiens |
|  | Mus musculus |
|  | Rattus norvegicus |

---

|  |  |
| --- | --- |
| Mouse chromosome position | 12 15.78 cM |
|  | 12 18.0 cM |

---

|  |  |
| --- | --- |
| OMIM ID | 600253 |

---

|  |  |
| --- | --- |
| Rat chromosome position | 6q16 |

---

|  |  |
| --- | --- |
| Hugo ID | 348 |
|  | HGNC:348 |

---

|  |  |
| --- | --- |
| Human chromosome position | 7p21.1 |
|  | 7p15 |

---

|  |  |
| --- | --- |
| Swiss-Prot Accession | A0A024R9Z8 |
|  | P35869 |
|  | P35869.2 |
|  | Q3U5D9 |
|  | P30561 |
|  | P30561.3 |
|  | Q8R4S5.1 |
|  | Q8R4S6.1 |
|  | G3V6M2 |
|  | P41738.2 |
|  | A4D130 |
|  | Q13728 |
|  | Q13803 |
|  | Q13804 |
|  | Q8QZX6 |
|  | Q8R4S3 |
|  | Q99P79 |
|  | Q9QVY1 |
|  | Q8R4S5 |
|  | Q8R4S6 |
|  | P41738 |
|  | O88930 |
|  | O89105 |
|  | Q3U2B1 |
|  | Q6DLW6 |
|  | Q6DLW7 |
|  | Q6DLW8 |
|  | Q6DLW9 |
|  | Q6DLX0 |
|  | Q6DLX1 |
|  | Q6DLX2 |
|  | Q6DLX3 |
|  | Q6DLX4 |
|  | Q6LEC9 |
|  | Q683N8 |

---

|  |  |
| --- | --- |
| PIR ID | S59514 |
|  | A46266 |
|  | S58375 |

---

|  |  |
| --- | --- |
| GenBank ID | NC\_000007 |
|  | NM\_001621 |
|  | NP\_001612 |
|  | AC003075 |
|  | AH003424 |
|  | AAA92082 |
|  | AAA92083 |
|  | AAA92084 |
|  | CH236948 |
|  | EAL24281 |
|  | CH471073 |
|  | EAW93686 |
|  | EAW93687 |
|  | D31708 |
|  | D38044 |
|  | BAA07235 |
|  | HF547963 |
|  | CCO13674 |
|  | LC377520 |
|  | LC377521 |
|  | LC377522 |
|  | LC377523 |
|  | LC377524 |
|  | LC377525 |
|  | LC377526 |
|  | LC377527 |
|  | LC377528 |
|  | LC377529 |
|  | LC377530 |
|  | LC377531 |
|  | LC377532 |
|  | LC377533 |
|  | LC377534 |
|  | LC377535 |
|  | U60524 |
|  | AA329775 |
|  | AK130815 |
|  | AK307495 |
|  | BC021696 |
|  | BC069390 |
|  | AAH69390 |
|  | BC070080 |
|  | AAH70080 |
|  | BQ020427 |
|  | BQ024331 |
|  | BX505160 |
|  | D16354 |
|  | BAA03857 |
|  | L19872 |
|  | AAA16210 |
|  | P35869 |
|  | NC\_000078 |
|  | NM\_001314027 |
|  | NP\_001300956 |
|  | NM\_013464 |
|  | NP\_038492 |
|  | AC159635 |
|  | AF325111 |
|  | AAK13443 |
|  | AH003146 |
|  | CH466526 |
|  | EDL36863 |
|  | M94623 |
|  | AAA02896 |
|  | S76844 |
|  | AAB33978 |
|  | U50336 |
|  | AF405560 |
|  | AAL89725 |
|  | AF405561 |
|  | AAL89726 |
|  | AF405562 |
|  | AAL89727 |
|  | AF405563 |
|  | AAL89728 |
|  | AF405565 |
|  | AAL89730 |
|  | AF405566 |
|  | AAL89731 |
|  | AF405567 |
|  | AAL89732 |
|  | AF405568 |
|  | AAL89733 |
|  | AF405570 |
|  | AAL89735 |
|  | AK014512 |
|  | AK136072 |
|  | AK153678 |
|  | BAE32140 |
|  | AK155380 |
|  | BAE33231 |
|  | AK171945 |
|  | AK172436 |
|  | AY662080 |
|  | AAT75227 |
|  | AY662081 |
|  | AAT75228 |
|  | AY662082 |
|  | AAT75229 |
|  | AY662083 |
|  | AAT75230 |
|  | AY662084 |
|  | AAT75231 |
|  | AY662085 |
|  | AAT75232 |
|  | AY662086 |
|  | AAT75233 |
|  | AY662087 |
|  | AAT75234 |
|  | AY662088 |
|  | AAT75235 |
|  | AY662089 |
|  | AAT75236 |
|  | AY662090 |
|  | AAT75237 |
|  | BC040248 |
|  | D38416 |
|  | P30561 |
|  | Q8R4S5 |
|  | Q8R4S6 |
|  | NC\_005105 |
|  | XM\_017594050 |
|  | XP\_017449539 |
|  | NM\_001308255 |
|  | NP\_001295184 |
|  | NM\_013149 |
|  | NP\_037281 |
|  | NM\_001308254 |
|  | NP\_001295183 |
|  | NR\_131776 |
|  | NR\_131775 |
|  | AC\_000074 |
|  | AABR07063901 |
|  | AAHX01043426 |
|  | AAHX01043427 |
|  | CH473947 |
|  | EDM03301 |
|  | EDM03302 |
|  | EDM03303 |
|  | EDM03304 |
|  | AF082124 |
|  | AAC35168 |
|  | AF082125 |
|  | AAC35169 |
|  | AF082126 |
|  | AAC35170 |
|  | AJ821851 |
|  | CAH25391 |
|  | AM902281 |
|  | CAP16657 |
|  | AM902282 |
|  | CAP16658 |
|  | AM902283 |
|  | CAP16659 |
|  | AM902284 |
|  | CAP16660 |
|  | AM902285 |
|  | CAP16661 |
|  | AM902286 |
|  | CAP16662 |
|  | U09000 |
|  | AAA56897 |
|  | P41738 |
|  | NC\_018918 |
|  | AMYH02015311 |
|  | AC\_000034 |
|  | AAHY01099184 |
|  | L19749 |
|  | L19750 |
|  | L19751 |
|  | L19752 |
|  | L19753 |
|  | L19754 |
|  | L19755 |
|  | L19756 |
|  | L19757 |
|  | AAA72026 |
|  | L19758 |
|  | L19759 |
|  | BC152973 |
|  | AAI52974 |
|  | BC156629 |
|  | AAI56630 |
|  | AC\_000068 |
|  | AC\_000139 |
|  | U28063 |
|  | U28064 |
|  | U28066 |
|  | NT\_079592 |
|  | NT\_007819 |
|  | NW\_001839003 |
|  | NW\_923240 |
|  | AC\_000050 |
|  | NT\_039548 |
|  | NW\_001030500 |
|  | Q3U2B1 |
|  | Q3U5D9 |
|  | Q6DLW6 |
|  | Q6DLW7 |
|  | Q6DLW8 |
|  | Q6DLW9 |
|  | Q6DLX0 |
|  | Q6DLX1 |
|  | Q6DLX2 |
|  | Q6DLX3 |
|  | Q6DLX4 |
|  | Q6LEC9 |
|  | NW\_001084845 |
|  | NW\_047760 |
|  | Q683N8 |
|  | ABBA01056640 |

---

|  |  |
| --- | --- |
| Swiss-Prot ID | AHR\_HUMAN |
|  | AHR\_MOUSE |
|  | AHR\_RAT |
|  | Q3U5D9\_MOUSE |
|  | A4D130\_HUMAN |

---

|  |  |
| --- | --- |
| Cell Localization | Cytoplasm |
|  | Nucleus |

---

|  |  |
| --- | --- |
| Ensembl ID | ENSG00000106546 |
|  | ENSP00000242057.4 |
|  | ENST00000242057.9 |
|  | ENSMUSG00000019256 |
|  | ENSMUSP00000112137.2 |
|  | ENSMUST00000116436.8 |
|  | ENSRNOG00000004342 |
|  | ENSRNOP00000006618.5 |
|  | ENSRNOT00000006618.7 |
|  | ENSP00000242057 |
|  | ENST00000242057 |
|  | ENSMUSP00000112137 |
|  | ENSMUST00000116436 |
|  | ENSRNOP00000006618 |
|  | ENSRNOT00000006618 |

---

|  |  |
| --- | --- |
| MGI ID | MGI:105043 |
|  | 105043 |

---

|  |  |
| --- | --- |
| RGD ID | 2074 |

---

|  |  |
| --- | --- |
| Unigene ID | Hs.171189 |
|  | Mm.341377 |
|  | Rn.91370 |

---

|  |  |
| --- | --- |
| Homologene ID | 1224 |

---

|  |  |
| --- | --- |
| Shape | O-vertex |

---

|  |  |
| --- | --- |
| IPI ID | IPI00131277 |
|  | IPI00231916 |
|  | IPI00364957 |
|  | IPI00021008 |
|  | IPI00625415 |
|  | IPI00890036 |

---
